# *Aspergillus awamori:* potential antioxidant, anti-inflammatory, and anti-apoptotic activities in acetic acid-induced ulcerative colitis in rats

**DOI:** 10.1007/s10787-024-01489-w

**Published:** 2024-05-20

**Authors:** Hoda A. Abd-Ellatieff, Kristen Georg, Abdel-Rahman. A. Abourawash, Emad. W. Ghazy, Dalia. H. Samak, Wael. M. Goda

**Affiliations:** 1 Pathology Department, Faculty of Veterinary Medicine, Damanhour University, Damanhour, Egypt; 2 Cure Lab Clinical Pathology, Kafrelsheikh University, Kafr El-Sheikh, Egypt; 3 Clinical Pathology Department, Faculty of Veterinary Medicine, Kafrelsheikh University, Kafr El-Sheikh, Egypt; 4 Department of Forensic Medicine and Toxicology, Faculty of Veterinary Medicine, Damanhour University, Damanhour, Egypt; 5 Clinical Pathology Department, Faculty of Veterinary Medicine, Damanhour University, Damanhour-El-Beheira, Egypt

**Keywords:** *Aspergillus awamori*, Colitis, Oxidative stress, Inflammatory mediators, Anti-oxidant, Apoptosis

## Abstract

Ulcerative colitis (UC) is a chronic colonic inflammation with a significant health hazard. *Aspergillus awamori (A. awamori*) is a microorganism with various bioactive compounds with natural antioxidant and anti-inflammatory properties. The present work aimed to elucidate the protective and therapeutic effects of varying concentrations of *A. awamori* against acetic acid (AA)-induced ulcerative colitis (UC) in rats. Nine groups of albino male rats were established: a control negative group (G1), a control positive group (G2,AA), and preventive protocol groups (including G3A, G4A, and G5A) that received 100 mg, 50 mg, and 25 mg/kg b.w, respectively, of *A. awamori* orally and daily from the 1st day of the experiment and for 7 consecutive days. Then, they were subjected to one dose of AA intrarectally on day 8th. G3B, G4B, and G5B were termed as curative protocol groups that received one dose of AA on day 8th and then administered 100 mg, 50 mg, and 25 mg/kg b.w. of *A. awamori,* respectively, on day 9th and continued receiving these doses daily until day 16th. Rats in the AA group exhibited marked histopathological alterations of the distal colon, with an exaggeration of the DAI. In addition, a remarkable increase in oxidative stress was represented by the elevation of MDA and NO levels with a decline in SOD and GPx activities. In addition, upregulation of TNF-α, IL-6, and IL-1β mRNA expressions and downregulation of* Muc2* and *Nrf2* levels were detected. Unambiguously, a remarkable anti-inflammatory effect was noticed either in *A. awamori* prevented or treated groups expounded by reducing and regulating TNF-α, IL-6, and IL-1β with improved pathological lesion scoring. The *Muc2*, *Nrf2*, and *bcl-2* gene levels were upregulated and restored also. In summary, the findings in this work reveal that *A. awamori* supplementation successfully alleviated the UC induced by AA, which had a better effect when administered before colitis induction.

**Supplementary Information:**

The online version contains supplementary material available at 10.1007/s10787-024-01489-w.

## Introduction

Ulcerative colitis (UC) and Crohn’s disease (CD) are chronic diseases of inflammatory bowel disease (IBD) that affect life quality significantly (Khor et al. 2011). Persistent ulcerative inflammation of the distal colon and rectal mucosa is known medically as UC (Podolsky 2002), which has increased gradually in recent years all over the world (Ananthakrishnan et al. 2022; Iskandar et al. 2015). UC is specified by ulceration, edema, bleeding, and infiltration of the intestinal mucosa by inflammatory cells with abscess formation in the mucosal crypts (Danese and Fiocchi 2011.). However, the causative agent of IBD has been widely studied during the last several decades, and the exact etiology of IBD occurrence and pathogenesis remains unclear. Genetic, environmental, and immune factors may be incorporated (Fiocchi 1998; Loftus Jr 2004).

Elevation of oxidative stress with over production of reactive oxygen species (ROS) and pro-inflammatory chemokines are essential factors implicated in the IBD pathogenesis and intestinal illness (Ardizzone and Bianchi Porro 2005; Nakamura et al. 2006). Tissue injury arises due to the uncontrolled and excessive activation of the immune response and elevated levels of nitrogen and oxygen metabolites (Pavlick et al. 2002). The reduction in the level of antioxidant enzymes with upregulation of oxidative stress leads to loss of mucosal barrier integrity, increased intestinal permeability, apoptosis, activation of NF-kB, and eventually colonic inflammation (Elblehi et al. 2021; Molodecky and Kaplan 2010). Crucially, apoptosis and extensive inflammatory cytokines, besides inflammatory cell infiltration, have been incorporated into IBD pathophysiology (Becker et al. 2013; Raish et al. 2021). INOS and COX-2 increase when inflammatory cytokines or microorganisms stimulate host cells, indicating their role in UC (Nussler and Billiar 1993; Simon 1999).

Until now, there is no straightforward remedy for UC and IBD treatment, as there is difficulty in controlling the exaggerated response of the immune system and oxidative stress (Aleisa et al. 2014). However, corticosteroids, anti‑TNF‑α antibodies, and immunosuppressive antibiotics, such as adalimumab, certolizumab, and infliximab, are drugs used in IBD treatment (Ardizzone et al. 2010). Unfortunately, the available therapies for IBD are characterized by their high costs, numerous side effects, and lack of effectiveness (Sartor 2004). Therefore, growing interest in finding safe, natural products with potent anti-inflammatory and antioxidant properties to treat UC has been developed recently (Shahid et al. 2022; Wang et al. 2019).

The antioxidant treatment approach has been used recently as a hopeful remedy for treating various diseases resulting from the imbalance between oxidant and antioxidant mechanisms (Baiseitova et al. 2023; Habotta et al. 2023).

*A. awamori* is one of the microorganisms that have various bioactive compounds of natural antioxidant and anti-inflammatory properties such as p-coumaric acid, gallic acid, and ascorbic acid (Salar et al. 2017), nigragillin, dyramide, and 5-caboxybenzofuran (Lotfy et al. 2019). It is considered a robust probiotic microorganism that has been utilized in food processing (Bigelis and Lasure 1987; Yokoyama et al. 2001), as it is used in citric acid production (Kadooka et al. 2019, 2020). In addition, *A. awamori* has many enzymes that enhance molecule digestion (Arora and Chandra 2010; Gracia et al. 2003). In addition, (Kanauchi et al. 2008)) reported that *A. awamori* produces a natural substance named feruloyl esterase, which is considered an antioxidative substance. Moreover*, A. awamori* alleviated lactose intolerance, hypocholesterolemia, and gastrointestinal illness (Delcenserie et al. 2008; Ljungh and Wadstrom 2006). Additionally, *A. awamori* has been reported to be effective against hepatic carcinoma (Assar et al. 2021) and cardiac and renal damage (Assar et al. 2022), with the enhancement of growth efficiency, immune response, nutrient digestibility, and reduction of lipid peroxidation in skeletal muscle (El‐Deep et al. 2021; Saleh et al. 2011, 2012).

All of the benefits mentioned above of *A. awamori* prove the fungi’s antioxidant, anti-inflammatory, and anti-microbial properties. Therefore, the current study was outlined to evaluate the preventive and curative action of *A. awamori* extract against AA-induced UC in male albino rats via assessing the hematological parameters, histopathological lesion scoring, apoptosis, and oxidative and inflammatory biomarkers.

## Materials and methods

### *A. awamori* extract preparation

*A. awamori* powder was brought from Kagoshima University, Faculty of Agriculture, Kagoshima, Japan. The preparations and administration of *A. awamori* were mentioned before (Assar et al. 2021). *A. awamori* powder was dissolved in saline at a concentration of 0.5 mg/mL as a stock solution, and the same concentration was used each time. The *A. awamori* extract was analyzed phytochemically using Ultra-Performance Liquid Chromatography (UPLC), as reported previously (Assar et al. 2021; Salar et al. 2017).

### Biological activity of *A. awamori* extract

#### Anti-oxidant activity

##### The 2,2-diphenyl-1-picrylhydrazyl (DPPH) assay

The free-radical scavenging potential activity (FRSP) of *A. awamori* extract was evaluated using the DPPH (2,2-diphenyl-1-picrylhydrazyl) radical scavenging assay as outlined earlier by (Salar et al. 2016) with a few modifications. A microtiter plate reader (BioTek Elx808, USA) was used according to the manufacturer’s guidelines to determine the changes in the absorbance at 517 nm for 0 to 30 min. The FRSP (%) was calculated using the equation

$${\mathrm{FRSP}}\, = \,\left( {{\text{control absorbance }}\left( A \right)\, - \,{\text{extract }}A} \right) \, / \, \left( {{\text{control }}A} \right)\, \times \,{1}00.$$

#### Animal management and experimental design

Eighty (*n* = 80) male albino rats, 8–10 weeks old and weighing 150–170 g, were purchased from the Laboratory Animal Research Center, Dokki, Egypt. Fresh and clean water was provided ad libitum to rats in separate metal cages. Rats were kept in the same habitat throughout the trial and fed the same diet. Before the experiment began, the animals were given 7 days of acclimatization. Animal pain and suffering were kept to a minimum as possible. The rats were randomly divided into nine groups, as seen in supplementary Fig. 1. The first group (G1, which served as a normal negative control, NC), comprised of 5 rats, received saline orally for 16 days (the duration of the experiment), with a single rectal instillation of saline on the 8th day of the experiment. The second group (G2, positive control group, acetic acid group = AA group) included ten rats receiving a single dose of 5% acetic acid (1 mL/rat) intrarectally on the 8th day. G3A, G4A, and G5A, termed prophylactic (preventive groups), and G3B, G4B, and G5B, termed treated or curative groups, have ten rats in each group. Groups 3A, 4A, and 5A received 100 mg, 50 mg, and 25 mg/kg b.w. orally of *A. awamori* aqueous solution (dissolved in saline)/daily by stomach tube for 7 consecutive days and then subjected to one dose of AA intrarectally at day 8th of the experiment. However, the curative groups received one dose of AA intrarectally on day 8th of the experiment. They then orally administered 100 mg, 50 mg, and 25 mg/kg b.w. of *A. awamori,* respectively, on the 9th day and continued receiving *A. awamori* extract until the 16th day (the end of the experiment). Group 6, comprised of 5 rats, received a single dose of acetic acid intrarectally on day 8th and then received 500 mg/kg of sulphasalazine (SSZ) orally on the 9th day.

### Colitis induction

Intrarectal infusion of 5% AA (1 ml) in saline solution was used to induce UC in rats. Soft 6F polypropylene catheters were used to enter the rectum at a depth of 4 cm inside. Rats were put in an inverted Trendelenburg position for 2 min during rectal installation to prevent leakage of intracolonic solution (Bezerra et al. 2017; Shahid et al. 2022). Xylazine and ketamine were used for rat anesthesia (5 mg/kg, i.p.).

### Assessments of colitis

#### Disease activity index (DAI)

Body weight loss scoring, stool consistency, and bloody stool percentage were calculated according to (Cooper et al. 1993), taking the score from 0 to 4. However, 10 cm of the distal part of the colon was excised, washed, opened and the feces were cleared by saline. Ratios of colon length to weight were evaluated.

#### Gross pathology and macroscopic lesion scoring

On the 16th day, a necropsy was executed immediately after euthanasia. The lower third of the colon was cleaned, weighed, and split longitudinally. Macroscopic lesion scoring for colitis was performed as reported previously (Morris et al. 1989). The macroscopic colon lesion was rated (0–5). 0 = negative visible changes; 1 = no ulcers with focal hyperemia; 2 = ulcers without noteworthy inflammation; 3 = liner ulcer with low inflammation; 4 = focal ulceration and inflammation; and 5 = diffuse inflammation and ulcerations.

### Colitis histopathological lesion scoring

Colonic tissue specimens were fixed in 10% neutral buffered formalin. Samples were rinsed, dehydrated in ethyl alcohol, cleared in xylene, and then embedded in paraffin. The paraffin Sects. (4 μm) were stained using hematoxylin and eosin (H&E) (Bancroft and Gamble 2008). As previously stated, the microscopic colon lesions were scored (Galvez et al. 2001; Gonzalez–Rey et al. 2006) on a 0–5 scale as follows: 0 = normal tissue; 1 = low ulceration with few leukocyte infiltration; 2 = mucosal and submucosal inflammation as well as mild leukocyte infiltration with focal or diffuse ulceration; 3 = mucosal, submucosal, and muscular inflammation with focal or diffuse ulceration along with moderate leukocyte infiltration; 4 = mucosal, submucosal, muscular, serosa inflammation, and high leukocyte infiltration with focal or diffuse ulceration; and 5 = inflammation included all layers of mucosa, submucosa, muscular, serosa, and transmural with diffuse extensive ulceration and transmural leukocyte infiltration.

### Blood indices

Blood samples were obtained in dry, clean EDTA-coated vacutainer tubes via ocular vein puncture. Exigo®, a wholly automated hematology analyzer (Boule Medical AB, Sweden), was employed in the hematological analysis.

### Tissue oxidative stress and antioxidant activity assay

The malondialdehyde (MDA) and nitric oxide (NO) levels were evaluated using a colorimetric assay kit (Biodiagnostic, Co., Dokki, Egypt) according to (Buege and Aust 1978) and (Miranda et al. 2001), respectively. The antioxidant enzymatic biomarkers, including superoxide dismutase (SOD) and glutathione peroxidase (GPx), were evaluated in colon tissues as described earlier (Marklund and Marklund 1974; Paglia and Valentine 1967) using colorimetric kits (Biodiagnostic, Co, Dokki, Egypt) in regarding to the manufacturer’s instructions.

### Apoptosis assay

According to the manufacturer’s instructions, caspase-3, bax, caspase-9, and *bcl*-2 levels were estimated using ELISA kits (CUBIO, Houston, TX, USA). Each sample was analyzed in triplicate.

#### Quantitative real-time polymerase chain reaction (qRT-PCR) for gene expression

Colon total RNA was extracted using the AllPrep DNA/RNA/Protein Mini Kit, Qiagen. The cDNA was obtained using the QuantiTectR Reverse Transcription Kit, Qiagen. qRT-PCR was done using (SYBR Premix Ex Taq II (Tli RNaseH Plus, Takara Bio) using the primer sequences listed in supplementary Table 1 for estimation of interleukin-1 Beta (IL-1β), interleukin-6 (IL-6), tumor necrosis alpha (TNF-α), mucin (*Muc2*), and the nuclear factor (erythroid-derived 2)-like 2 (*Nrf2*). The housekeeping β-actin was used to normalize the value of each examined sample. The relative changes between samples were conducted using the 2^−∆Ct^ method (Livak and Schmittgen 2001).

### Data analysis

The normality and homogeneity between all the data were determined using the Shapiro–Wilk test. A one-way ANOVA followed by Duncan’s *post hoc* test was used to compare the significance between groups using SPSS v.17 statistics. Values that are *P* < 0.05 are statistically significant between groups. Data were expressed as mean ± standard error (SEM).

## Results

### Biological activity of *A. awamori* extract

#### Anti-oxidant activity (DPPH assay)

*A. awamori* displayed robust radical scavenging activity using the DPPH assay with 69.32% as compared to 54.75%, and 29.65% for ascorbic acid and catechin respectively. This value potentially determining the effectiveness of *A. awamori* in neutralizing free radicals in regard to the commonly known antioxidants such as ascorbic acid and catechin.

#### Bioactive compounds of *A. awamori* extract using UPLC analysis

**Fig. 1:**
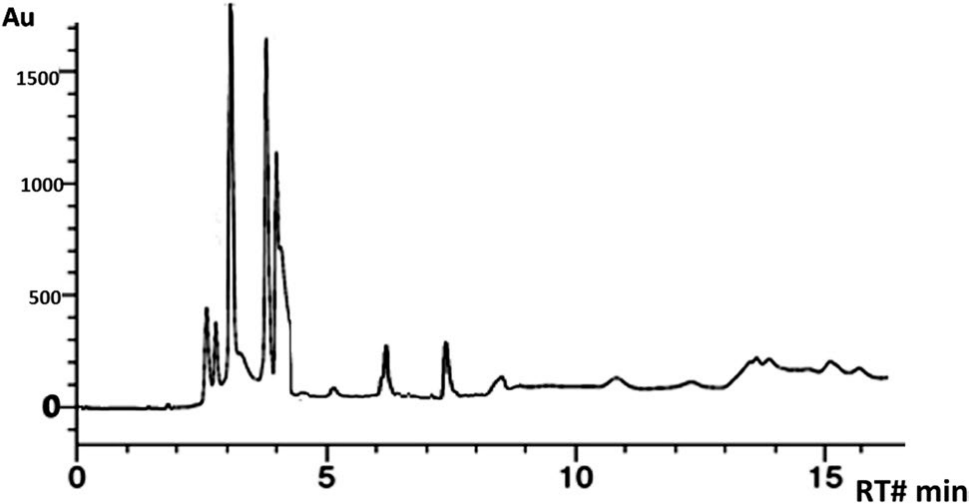
UPLC chromatogram of *A. awamori* aqueous ethanol extract

The bioactive compounds identified are p′-Coumaric acid, citric acid, ascorbic acid, gallic acid, and gentisic acid and there are many compounds that of unknown details and need further analysis. These compounds were shown in Fig. 1 and supplementary Table 2. The presence of such compounds reveal the biological action potential of *A. awamori* in our work.

#### Clinical signs and DAI expressed by experimental animals

Rats inoculated with AA in G2 exhibited severe clinical signs of severe loss in body weight, diarrhea, and bloody stool with mucus compared to the NG group (G1). On the contrary, rats received *A. awamori* extract (100 mg, 50 mg, and 25 mg/kg b.w.) either before induction of colitis (G3A, G4A, and G5A) or after colitis induction (G3B, G4B, and G5B), appeared clinically normal, and exhibited mild clinical signs of abdominal pain and diarrhea. No clinical signs were seen either in the NC or the sulphasalazine-treated (SSZ) groups (Table 1).

**Table 1:** Clinical signs and Disease activity index (DAI) expressed by experimented animals

Groups	Initial weight	Final weight	Stool consistency Score	Blood in stool	Colon width	Colon length	Rectal bleeding	DAI Score
G1 (negative control, NC)	121 ± 4.3	166.2 ± 7.97^a^	0 ± 0.0^d^	0 ± 0^f^	1.03 ± 0.02^e^	13.86 ± 0.38^a^	Normal	0.00 ± 0.00
G2 (Acetic acid, AA)	122.2 ± 2.91	142.4 ± 8.70^d^	3.3 ± 0.14^a^	2.4 ± 0.75^a^	1.78 ± 0.14^a^	8.66 ± 0.48^d^	Occult + + + +	4 ± 0.10
G3 A	123.15 ± 2.6	149.6 ± 5.94^c^	1.6 ± 0.15^b^	1.69 ± 0.12^c^	1.40 ± 0.12^c^	12.01 ± 0.15^b^	Occult + +	1.90 ± 0.12
G4 A	125 ± 3.15	164.2 ± 3.31^b^	1.0 ± 0.25^c^	0.79 ± 0.15^d^	1.19 ± 0.12^d^	12.58 ± 0.36^b^	Occult +	0.89 ± 0.11
G5 A	121.4 ± 3.31	162.2 ± 4.31^b^	o.8 ± 0.18^c^	1. 2 ± 0.14^b^	1.15 ± 0.1^b^	11.7 ± 0.25^c^	Occult + +	1.77 ± 0.19
G3 B	123.75 ± 2.6	148 ± 5.84^c^	1.5 ± 0.16^b^	1.7 ± 0.12^c^	1.42 ± 0.12^c^	12.21 ± 0.17^b^	Occult + +	1.86 ± 0.19
G4 B	125.3 ± 3.15	163.2 ± 3.31^b^	0.8 ± 0.15^c^	0.84 ± 0.12^d^	1.14 ± 0.1^b^	12.7 ± 0.15^c^	Occult +	0.94 ± 0.13
G5 B	122.2 ± 3.11	163.2 ± 4.21^b^	o.8 ± 0.18^c^	0.92 ± 0.14^b^	1.15 ± 0.1^b^	11.7 ± 0.25^c^	Occult +	1.77 ± 0.18
G6 (Sulphasalazine)	127.2 ± 3.61	164.2 ± 7.97^a^	o.6 ± 0.28^c^	0.32 ± 0.12^d^	1.06 ± 0.1^b^	12.9 ± 0.15^c^	Occult +	0.01 ± 0.62

Data expressed as means±SEM. The significant change was at p < 0.05. Means with in the same columns carrying the superscript letters (a,b,c,d) are significantly different (p<0.05)

DAI is used to evaluate colonic injury based on the symptoms exhibited by the experimental animal. As displayed in Table 1, a significant increase in DAI, expressed by a reduction in final body weight, bloody diarrhea, mucosal erosion, and rectal bleeding in AA-administered rats (G2) (scoring 4) in relevant to the NC group (G1) (*p* < 0.05). Moreover, the preventive (G3A, G4A, and G5A) and curative (G3B, G4B, and G5B) groups displayed a lower DAI score (Table 1) compared to the G2. Remarkably, the preventive (4A) group that received (50 mg/kg) showed a lower DAI score (*p* < 0.01) than the AA group (G2). The NG (G1) and sulphasalazine (G6) did not exhibit any signs of inflammation clinically, and the DAI score was less than ≺ 1 (*p* < 0.05), (Fig. 5e).

### Gross pathology

Grossly, AA-inoculated rats in G2 showed high macroscopic lesion scoring in the form of increased colon weight, colon hemorrhage, and ulceration. However, mild-to-moderate colon swelling, hemorrhage, and ulcerations were observed in the preventive and curative groups (G3A, G4A, G5A, G3B, G4B, and G5B). No visible damage was detected either in G1 or G6.

### Blood indices

The hematological parameters of all experimental groups are summarized in supplementary Table 3. An apparent decrease in hemoglobin (Hb) content, red blood cell (RBC) count, hematocrit (HCT), and mean corpuscular hemoglobin concentration (MCHC) were documented in the AA group (G2) in relation to the NC group. On the other hand, as shown in supplementary Table 4, a significant increase in the total count of leucocytes (WBCs), platelets, and mean platelet volume (MPV) was observed in the AA group (G2). The neutrophil count was the highest, while the lymphocyte count was the lowest, with no significant change in eosinophil, basophil, and monocyte counts. In contrast, rats treated with* A. awamori* (G3A, G4A,, G5A, G3B, G4B, and G5B), as well as those that received the standard sulphasalazine drug (G6), explained a significant decrease in total leucocyte (WBCs), platelets, MPV, and neutrophil count, with a significant increase in lymphocyte count. The best values were seen in rats who received the moderate dose of *A. awamori* (50 mg/kg bw), especially before induction of colitis.

### Oxidative and antioxidant marker assay

**Fig. 2:**
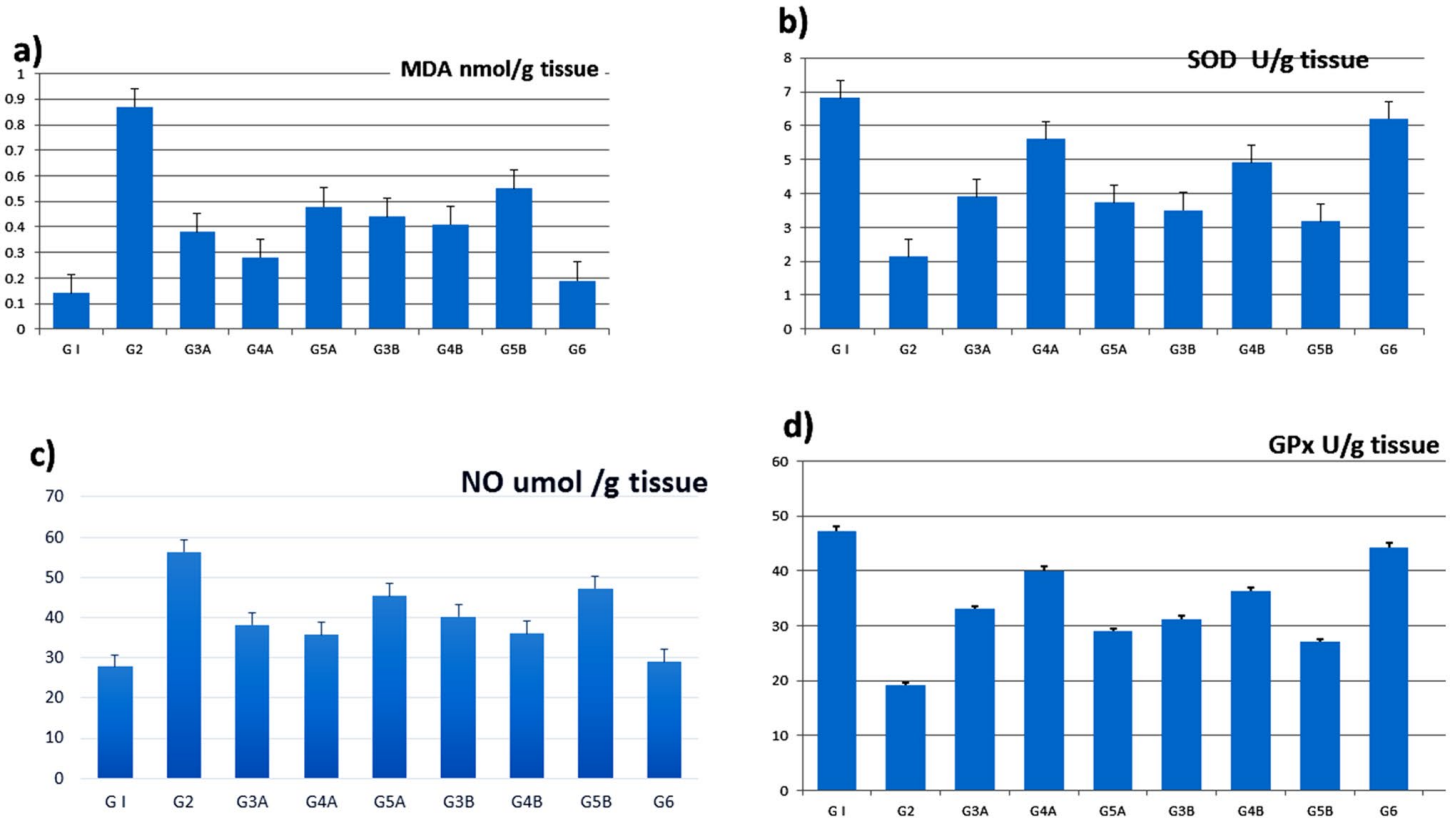
Mean values of colon MDA, SOD, NO, and GPX, in different experimented groups that are significantly different at (P ≤ 0.05). Data are presented as (Mean ± SEM), SEM = Standard error of the mean

As illustrated in Fig. 2, there was a significant upregulation in MDA and NO levels in the AA group with an apparent decrease in GPx and SOD levels, along with a downregulation of the *Nrf2* mRNA levels in the AA group (G2) relevant to the NC group (G1), revealing the enhancement of oxidative stress damage and lipid peroxidation. In contrast, a noticeable decrease in MDA and NO levels with a notable increase in GPx, SOD, and *Nrf2* levels were observed in the preventive (G3A, G4A, and G5A) and curative (G3B, G4B, and G5B) groups as compared to the AA group (G2). The administration of *A. awamori* before AA administration gave better-enhanced results, as the protective and boosting effect of *A. awamori* was shown to have a dose-dependent effect (Fig. 2).

### Inflammatory marker assay

**Fig. 3:**
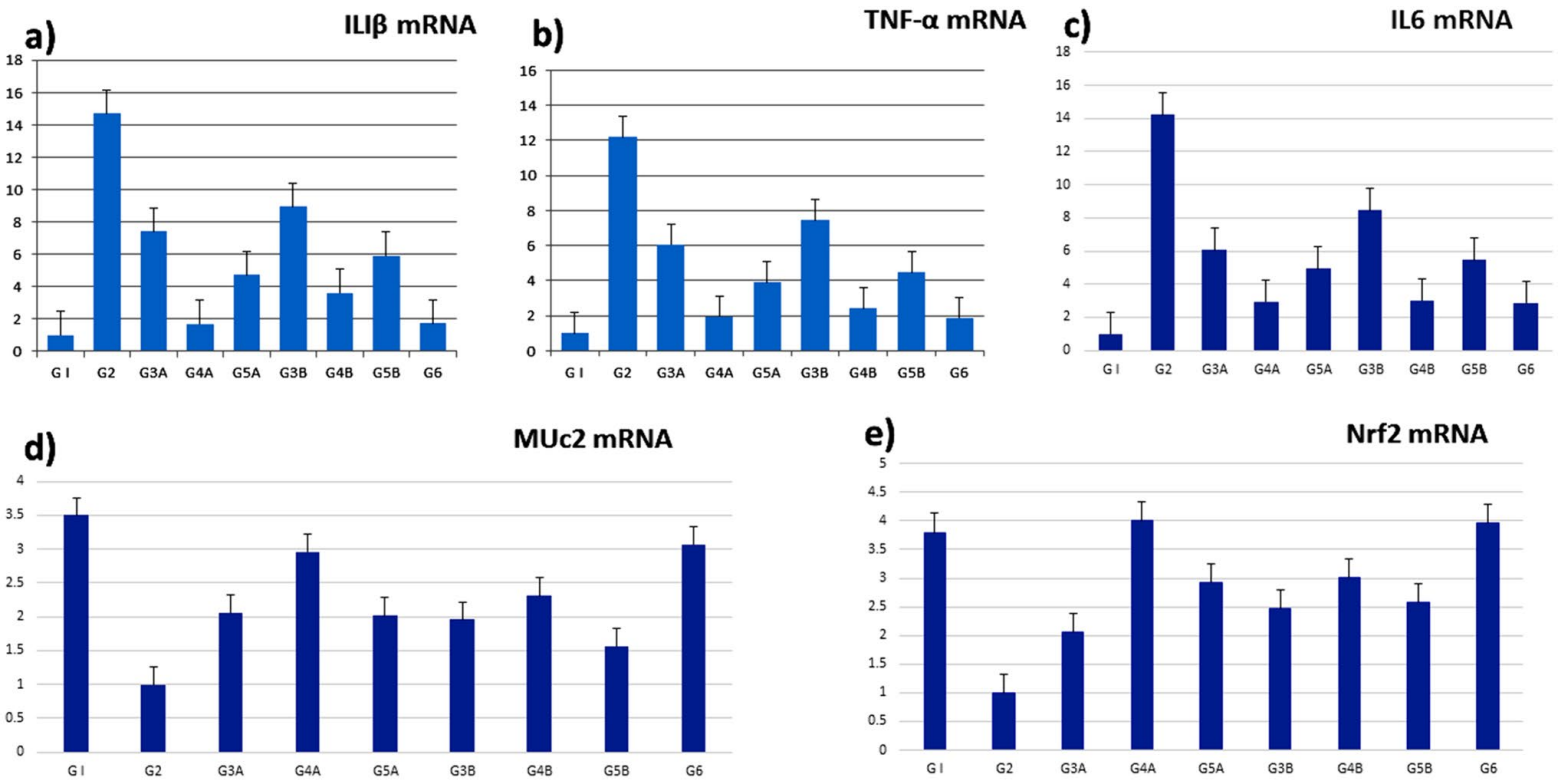
Changes in the relative expression of IL1β, TNFα, Muc2, IL 6, and NrF2 genes in the colon of administrated, preventive and curative groups. Values are significantly different at *p* < 0.05. Values are presented as means ± SEM

The rats in the AA (G2) exhibited severe UC expressed by a dramatic increase in IL-β, TNF-α, IL-6, and iNOS compared to the NC, revealing the triggering of inflammation. Upon administration of *A. awamori*, a significant decline (P < 0.001) in the expression of inflammatory cytokine genes was detected as compared to the AA (Fig. 3). Remarkably, the preventive groups displayed a better amelioration of UC.

### Effect of *A. awamori* on *Muc2*

A significant reduction (*p* < 0.001) in the *Muc2* gene was detected in AA (G2) as compared to NC (G1). Administration of *A. awamori* at dose-dependent values significantly upregulated and restored the levels of the *Muc2* gene in comparison to the AA. G4A represents the best-improved group (Fig. 3).

### Pro-apoptotic and anti-apoptotic profile pathways

As shown in Table 2, AA exhibited marked apoptosis in the colonic tissues, indicated by a substantial increase in the pro-apoptotic genes (bax, caspase-3, caspase-9), with a considerable reduction in the level of the anti-apoptotic protein (*bcl-2*) in comparison to the NC. In the preventive groups (G3A, G4A, and G5A) and curative groups (G3B, G4B, and G5B), the anti-apoptotic (*bcl-2*) gene was significantly upregulated (*p* ≤ 0.05), with a marked decline in the bax, caspase-3, and caspase-9 pro-apoptotic genes concerning AA (G2). Noteworthy, the preventive groups (G3A, G4A, and G5A) substantially modulated and restored the expression of the anti-apoptotic (*bcl-2*) gene, revealing low cell death. There were no significant alterations either in the sulphasalazine-treated group or the NC.

**Table 2:** Effect *of A. awamori* on pro-apoptotic and anti-apoptotic markers level in colon

Groups	Bcl-2	Bax	Caspase-3	Caspase-9
G1	14.87 ± 0.65^a^	2.7 ± 0.25^a^	1.68 ± 0.09^a^	5.21 ± 0.18^a^
G2	5.8 ± 0.78^b^	8.22 ± 0.32^b^	10.30 ± 0.67^b^	15.26 ± 0.89^b^
G3A	10.38 ± 0.43^c^	4.12 ± 0.53^c^	3.01 ± 0.28^c^	3.58 ± 0.46^c^
G4A	13.78 ± 0.75^a^	2.6 ± 0.23^a^	1.47 ± 0.08^a^	4.81 ± 0.19^a^
G5A	12.98 ± 0.78^a^	3.20 ± 0.32^a^	2.19 ± 0.19^a^	4.00 ± 0.43^a^
G3B	9.87 ± 0.47^c^	3.12 ± 0.56^c^	2.81 ± 0.26^c^	3.18 ± 0.46^c^
G4B	11.98 ± 0.65^a^	2.80 ± 0.22^a^	1.17 ± 0.07^a^	4.11 ± 0.28^a^
G5B	12.08 ± 0.58^a^	3.01 ± 0.62^a^	1.96 ± 0.54^a^	3.90 ± 0.25^a^
G6	14.57 ± 0.65^a^	2.6 ± 0.29^a^	1.58 ± 0.38^a^	5.00 ± 0.38^a^

Data expressed as means±SEM.The significant change was at p < 0.05. Means with in the same columns carrying the superscript letters (a,b,c,d) are significantly different (p<0.05)

### *A. awamori* ameliorated the histopathological lesions induced by AA in rats

**Fig. 4:**
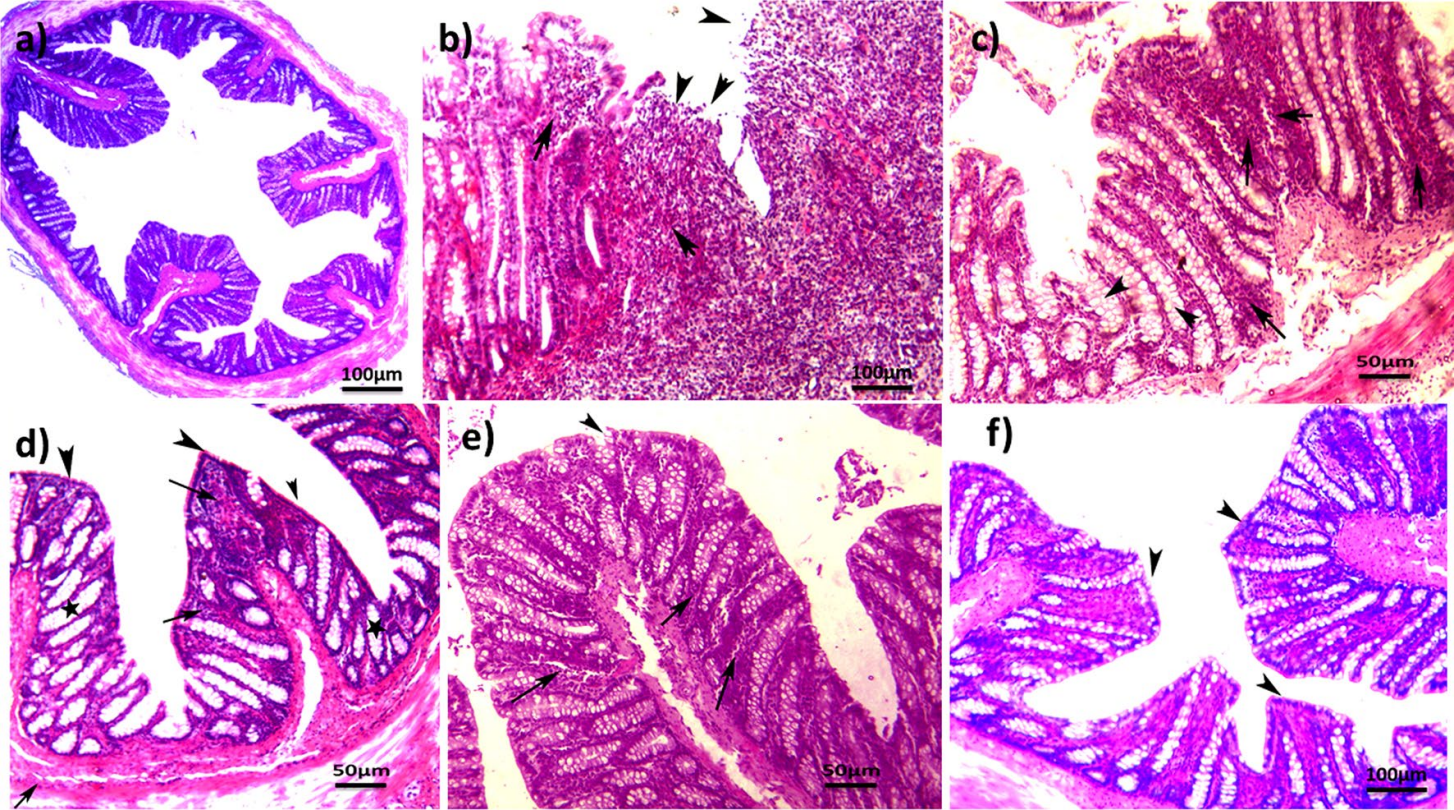
Histopathological photos stained by H&E. **a)** Control rats who received saline rectally showed normal architecture of mucosa, submucosa, and muscularis layer, with the intact epithelial surface. **b)** Rats received AA rectally characterized by severe colitis described in heavy infiltration of mucosal and submucosal layer by leukocytes (arrows) with sloughing of epithelial mucosa (arrowhead). **c–e)** preventive groups received (100, 50, and 25 mg/kg *A.awamori*) respectively. **c)** *A.awamori* preventive group received (100 mg/ kg) characterized by moderate colitis with moderate inflammatory cell infiltrations of mucosal and submucosal layers (arrows), goblet cell hyperplasia (arrowhead), and mild epithelial erosions. **d)** *A.awamori* preventive group received (50 mg/ kg) displayed mild colitis with few inflammatory cells (arrows) infiltration of mucosal and submucosal layer, intact epithelial mucosa (arrowhead), and mild goblet cell loss (black star). **e)** *A.awamori* preventive group received (25 mg/ kg) showed mild-to-moderate colitis with leukocytic cell infiltration (arrows), almost intact epithelial mucosa, and goblet cell hyperplasia (arrowhead). **f)** The sulphasalazine-treated group showed mild colitis with few inflammatory cells (arrows) infiltration (arrowhead) and intact epithelial mucosa (arrows)

**Fig. 5:**
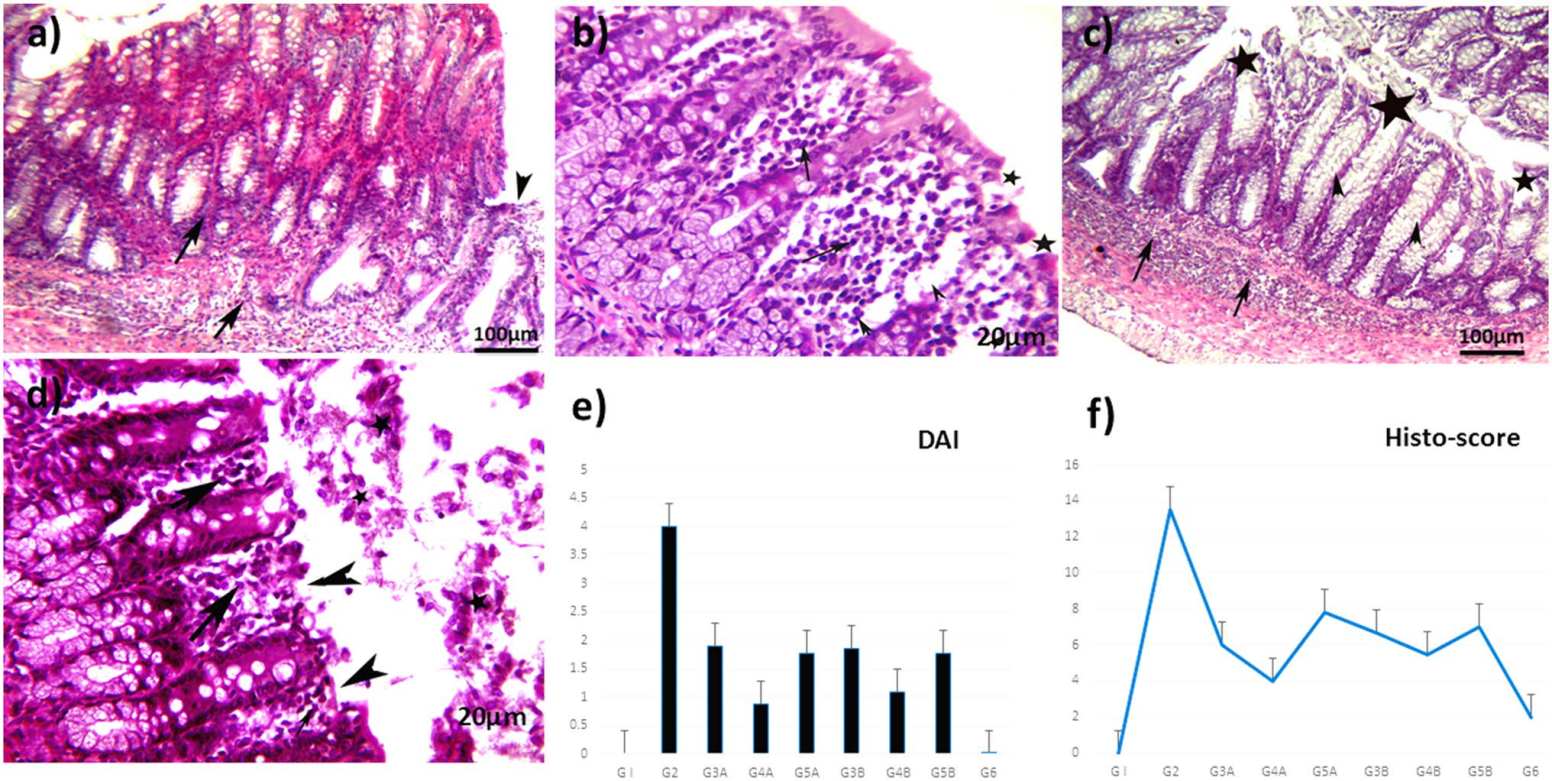
**a-d)** rats received AA rectally and then post-treated by (100, 50, and 25 mg/kg *A.awamori*) respectively. **a)** *A.awamori* curative group received (100 mg/ kg) characterized by (moderate +) colitis with inflammatory cell infiltrations of mucosal and submucosal layers (arrows), and mild erosion of epithelial mucosa (arrowhead). **b)** *A. awamori* curative group received (50 mg/ kg) displayed moderate colitis of leukocytic cell infiltrations (arrows), mild edema (arrowhead), with mild erosion of epithelial mucosa (black star). **c), d)** *A. awamori* curative group received (25 mg/ kg) showed moderate + colitis, infiltration of inflammatory cells (arrows) in the mucosal and submucosal layer, and goblet cell hyperplasia (arrowhead), with erosion of epithelial mucosa (black star). **d)** Higher magnification of previous photo showed moderate + colitis with leukocytic cell infiltration (arrows), erosion of epithelial mucosa (arrowhead), with epithelial desquamation (black star), H&E. **e)** DAI graph in experimented groups. Data are presented as (Mean ± SEM), SEM = Standard error of the mean. **f)** Histopathological lesion scoring between different experimented groups

As illustrated in Fig. 4, the AA group (G2) revealed serious microscopic damage scoring (score 5) compared to the NC group. Rats in the AA group exhibited inflammatory reactions in the form of moderate-to-severe mucosal and submucosal erosion, inflammatory cell infiltrations, distortion of cryptic architecture, ulcerations, and epithelial desquamation and necrosis (Fig. 4b). *A. awamori*, when given as a protective dose before induction of colitis in (G3A, G4A, and G5A) significantly attenuated the severity of the histopathological alterations and restored the colonic mucosa architecture. A very mild degree of colitis (score 2), low inflammatory cell infiltrations with few erosions, and no ulcers were detected in the examined tissues of the rats in that group (Figs. 4c–e). Few or no pathological alterations were detected in the sulphasalazine group (Fig. 4f), as the epithelial mucosa is intact with goblet cell proliferation. However, the *A. awamori* post-treated groups (G3B, G4B, and G5B) showed moderate improvement in colitis as compared to the acetic acid group. In contrast, the mucosal and submucosal layers showed mild-to-moderate colitis with few-to-moderate inflammatory cell infiltration, mild edema, slight erosions, and ulceration of mucosal columnar epithelial cells (Figs. 5a–d). The histopathological lesion scoring and DAI affirmed the role of *A. awamori* in protecting the colonic tissues from damage and their ameliorating effect against UC induced by AA (Fig. 5e and d).

## Discussion

IBD is a chronic inflammatory disease that negatively affects the patient’s health, causing severe gastrointestinal disturbances and may lead to colorectal cancer (Pandurangan and Esa 2014). Various factors and mechanisms, such as immunological, environmental, sex, and age, were involved in the IBD pathogenesis, but oxidative stress, with augmentation of free radicals and ROS generation, are the essential contributory factors for IBD occurrence (Bhattacharyya et al. 2014).

AA-induced UC resembles human UC concerning pathological sequences and histopathological alterations; thus, it is considered an effective tool for searching for and screening suitable safe drugs of anti-colitic activity (Randhawa et al. 2014; Shahid et al. 2022).

Herein, the consequences of the AA on the induction of UC and toxic damage of colonic tissues are displayed by a noteworthy upregulation of MDA and NO levels with a reduction of SOD and GPx antioxidant activity. Acute lipid peroxidation (LPO) and ROS overproduction were evidenced here by a substantial increase in MDA levels, disrupting the cell membrane permeability and integrity (Medicherla et al. 2015). Furthermore, NO, the nitrosative stress marker, enhances the formation of the pro-oxidant peroxynitrite after its reaction with the superoxide radical. The pro-oxidant peroxynitrite is further decomposed into the nitro radical, resulting in excessive nitrosative stress and DNA damage (Heikal et al. 2009). GPx is an antioxidant with a central role in ROS scavenging (Shahraki et al. 2023). Additionally, the SOD is a crucial and essential antioxidant defense enzyme against oxidative stress in the cell (Younus 2018). These findings are supported by previous related studies (Arab et al. 2014; Ju et al. 2018; Minaiyan et al. 2014; Nagib et al. 2013; Raish et al. 2021; Ran et al. 2008), which corroborated the role of increased MDA and LPO levels in the induction of extensive cellular damage and elevation of pathological lesion scoring in UC (Nagib et al. 2013; Soliman et al. 2022a; Witaicenis et al. 2012).

Oxidative stress results in the excess production of ROS, which further upregulates the release of pro-inflammatory and inflammatory cytokines (Owumi et al. 2020). Based on the investigation in the current study, AA considerably enhanced the levels of the pro-inflammatory cytokines (IL-1β, Il-6, and TNF-α) in the colonic tissue, which further promotes chemotaxis, chemokine release and triggers the inflammatory mediator’s cascade (Abd-Ellatieff et al. 2020; Monteleone et al. 2002; Shahraki et al. 2023; Shapiro et al. 2007; Stallmach et al. 1992). Moreover, the *Nrf2* mRNA level was upregulated. It is a transcription factor, typically present in the cytoplasm of cells, and promotes the reduction of oxidative stress bursts (Zhang and Wang 2018). *Nrf2* activates antioxidant enzymes such as SOD and GSH-Px (de Vries et al. 2008), which react with free radicals, minimize oxidative stress, and protect vital cells from damage by disrupting radicals’ activity. By binding to the promoter’s antioxidant gene response element (ARE), *Nrf2* stimulates the expression of antioxidant genes. According to the findings in this study, AA considerably declines the expression of the *Nrf2* gene, causing disruption of the *Nrf2*/ARE pathway and thus exaggerating oxidative stress and further tissue damage (de Vries et al. 2008; Itoh et al. 2004). Consequently, pathological outcomes vividly confirmed the UC by severe tissue damage in mucosal erosions, ulcerations, epithelial cell necrosis, neutrophil infiltration, and edema (Mizushima et al. 2009; Siegmund 2002). Furthermore, UC induced by AA was characterized by several clinicopathological alterations in experimental rats, such as bleeding, weight loss, colonic shortening with ulceration, edema, and erosions; however, these results agree with the previous studies (Randhawa et al. 2014; Shahid et al. 2022). In addition, the increase of the DAI in the colitic group was in line with the results of (Abdel-Daim et al. 2015; Akgun et al. 2005; Shahid et al. 2022).

Mitochondrial oxidative stress enhances and triggers apoptosis and other cellular signaling pathways. Apoptosis means the eradication of undesired cells by the death of those cells, which is achieved by intrinsic mitochondrial and extrinsic death receptor pathways (Venkatadri et al. 2016). Inflammatory responses induce alterations in the intestinal integrity and mucosal barrier function leading to apoptosis; therefore, it is a crucial factor in the pathophysiology of IBD (Ali et al. 2017). Apoptosis has been regulated by the anti-apoptotic (*bcl-2*) and pro-apoptotic (bax, caspase-3, and caspase-9) proteins. The ratio of pro-apoptotic to anti-apoptotic is one of the key factors that regulate apoptosis (Zhao et al. 2019). Bax regulates cytochrome c liberation inside the cytosol and enhances the mitochondrial-permeability transition (MPT) (Shalini et al. 2015), leading to caspase-9 activation, which cleaves caspase-3 (Kuida 2000) and eventually apoptosis occurs (Kaur et al. 2020). However, *bcl-2* suppresses the release of bax proteins and stabilizes the MPT, thus restricting the activation of apoptotic pathways. In addition, *bcl-2* prevents the discharge of cytochrome c into the cytosol, thus protecting the cell and increasing its longevity. Following these studies, investigations in this work proved the enhancement of apoptosis following AA administration by the significant increase of the pro-apoptotic (caspase-3, caspase-9, and bax) and downregulation of the anti-apoptotic (*bcl-2*) proteins.

*A. awamori* is enriched by various bioactive compounds of natural antioxidant and anti-inflammatory properties. *A. awamori*’s antioxidant mechanism demonstrates a beneficial effect against UC. Interestingly, results in this study revealed that *A. awamori,* either before or after induction of UC by AA in the preventive and curative protocol, respectively, could stretch a sturdy antioxidant action against the oxidative stress induced by AA. *A. awamori* supplementation can withstand the oxidative injury via LPO mitigation by reducing of MDA and NO levels. Additionally, *A. awamori* protected the colonic tissue from damage by restoring and boosting the antioxidant activity of SOD, GPx, and *Nrf2* to their normal grades as compared to the AA group. The preventive and curative aptitude of *A. awamori* in free-radical scavenging is attributed to its richness in flavonoids and polyphenolic compounds, such as p-coumaric acid, gallic acid, cinnamic acid, ascorbic acid (Salar et al. 2017), nigragillin, dyramide, 5-caboxybenzofuran (Lotfy et al. 2019), and feruloyl esterase (Kanauchi et al. 2008). Furthermore,* A. awamori* contains many enzymes that enhance molecule digestion (Arora and Chandra 2010; Gracia et al. 2003). Additionally, many studies have reported the effectiveness of* A. awamori* against hepatic carcinoma (Assar et al. 2021), cardiac and renal damage (Assar et al. 2022), and gastrointestinal illness (Delcenserie et al. 2008; Ljungh and Wadstrom 2006), depending upon the antioxidant activity of the* A. awamori*. Receiving *A. awamori* (especially the dose of 50 mg/kg) either before or after UC induction improved and ameliorated the pathological alterations and the DAI concerning the NC group. These results align with the previous studies where bioactive polyphenol compounds ameliorate AA-induced UC (Ju et al. 2018; Shapiro et al. 2007; Tahan et al. 2011).

The inflammatory cytokines (TNF-α, IL-β, and iNOS) are considered the earlier signs of inflammation, and controlling their levels and expressions is the target and goal for the proper treatment of any disease (Gillberg et al. 2013; Kitabatake et al. 2021; Liu and Wang 2011; Soliman et al. 2022b). In the current work, the elevated levels of IL-1β, IL-6, and TNF-α in the AA group were reduced and regulated upon *A. awamori* administration refers to the potent anti-inflammatory activity of *A. awamori* and its role in inhibiting the transcription of these cytokines, thus decreasing tissue damage (Islam et al. 2008; Shahid et al. 2022). Besides, the anti-apoptotic effect of *A. awamori* was elucidated by the increased levels of the anti-apoptotic *bcl-2* protein, either in preventive or curative groups, compared to the AA group, which prompted upregulation of pro-apoptotic proteins (caspase-3, bax, and caspase-9).

The gastrointestinal mucosa was protected from chemical, microbial, and mechanical damage via mucus, secreted by goblet cells as mucin and encoded by the *Muc2* gene (Willemsen et al. 2003). A significantly higher expression of the *Muc2* gene in colonic tissues in *A. awamori* treated (especially G4A) and NC groups compared to the AA group indicates that *A. awamori* maintains mucus production and protects colonic tissues from damage via suppressing inflammation-associated genes. The lower expression of *Muc2* in the colonic tissue, as in the AA, leads to abnormalities in tissue morphology, including increased gut mucosa thickness, inflammatory cell infiltration, and aggravation of UC pathogenesis (Islam et al. 2017).

Nevertheless, the administration* of A. awamori* (either as a preventive or curative dose) significantly reduced UC features and protected rats from severe colitis. The protection mechanism of *A. awamori* against UC could be attributed to the anti-inflammatory, antioxidant, and anti-apoptotic properties of *A. awamori*.

## Conclusion

The results of this study elucidated the preventive and curative impact of *A. awamori* against AA-induced UC in rats. However, this is likely attributed to the anti-inflammatory, antioxidant, and anti-apoptotic properties of *A. awamori*. Administration of A.* awamori* in rats either before or after colitis induction maintained the colonic tissue’s architecture, function, and integrity. A notable improvement in the antioxidant defense was achieved by the upregulation of SOD, GPx, and *Nrf2* and the decline of MDA and NO levels. Besides, a marked reduction in the pro-inflammatory cytokines and increased anti-apoptotic (*bcl-2*) protein were noticed. Furthermore, *A. awamori* supplementation significantly ameliorates DAI and the tissue damage of AA-induced UC. Therefore, further investigations for developing a strategy using *A. awamori* as a therapeutic and prophylactic product against IBD should be considered.

## Supplementary Information

Below is the link to the electronic supplementary material.

Supplementary file1 (DOCX 21 KB)

Supplementary file2 (TIF 1032 KB)

## Data Availability

All data sets obtained and analyzed during the current studyare available in the manuscript. Further inquiries can be directed to the corresponding author.

## Abbreviations

*A. awamori*: Aspergillus awamori
DAI: Disease Activity Index
GPx: Glutathione peroxidase
IL1β: Interleukin-1bet
IL6: Interleukin 6
NO: Nitric oxide
MDA: Malondialdehyde
Muc2: Mucin
Nrf2: Nuclear factor erythroid 2-related factor 2
PG: Prostaglandins
SOD: Superoxide dismutase
TNF-α: Tumor necrosis alpha
