# Supplementary material for: Aspergillus awamori: potential antioxidant, anti-inflammatory, and anti-apoptotic activities in acetic acid-induced ulcerative colitis in rats
Source: Inflammopharmacology. 2024 May 20;32(4):2541–53. doi: 10.1007/s10787-024-01489-w (PMC11300502; doi:10.1007/s10787-024-01489-w)
Supplement: Supplementary file 1 — Supplementary file1 (DOCX 21 KB) [file 10787_2024_1489_MOESM1_ESM.docx]

Supplementary Table1. Forward and reverse primers sequence for IL1β, TNF α, IL6, Nrf2, Muc2 and β-actin genes

| **Gene** | **Forward primer** | **Reverse primer** | **Accession number** |
| --- | --- | --- | --- |
| IL1β | CACCTCTCAAGCAGAGCACAG | GGGTTCCATGGTGAAGTCAAC | NM_031512.2 |
| TNF-α | GCATGATCCGCGACGTGGAA | AGATCCATGCCGTTGGCCAG | X66539.1 |
| IL6 | CCCACCAGGAACGAAAGTCA | ACTGGCTGGAAGTCTCTTGC | M26744.1 |
| Nrf2 | TGTCAGCTACTCCCAGGTTG | ATCAGGGGTGGTGAAGACTG | NM_031789.2 |
| Muc2 | CAAGGTCTACTCCCGTGCTG | GAGGAGTCCTACCACCCCAG | U68172 |
| β-actin | AGGGAAATCGTGCGTGAC | CGCTCATTGCCGATAGTG | EF156276.1 |

Supplementary Table 2. Bioactive compounds of *A. awamori* using UPLC in aqueous ethanol extract.

| RT# | Compound | Concentration  mg/g |
| --- | --- | --- |
| 3.4 | p′-Coumaric acid | 5.96 |
| 6.5 | gallic acid | 2.27 |
| 7.0 | ascorbic acid | .98 |
| 4.3 | Cinnamic acid | 4.31 |
| 6 | Benzoic | 1.61 |
| 4 | Citric acid | 3.74 |
| 7.5 | Gentisic acid | 1.88 |
| 8.6 | 1,5-dimethyl citrate | 0.6 |
| 10.8 | methyl salicylate | 0.8 |

Supplementary Table 3. Hematological parameters in different experimented groups

| **Group** | **Hb**  **(g/dl)** | **RBCs**  **(10^6^/µl)** | **HCT**  **(%)** | **MCV**  **(fl)** | **MCH**  **(fl)** | **MCHC**  **(fl)** | **RDW**  **(fl)** |
| --- | --- | --- | --- | --- | --- | --- | --- |
| **G1** | 14.2±0.54 **^a^** | 4.26±0.24 **^a^** | 44.8±1.03 **^a^** | 70.48±1.65 **^c^** | 23.82±0.76 **^c^** | 33.1±0.87 **^a^** | 15.88±0.73 |
| **G2** | 10.15±0.41 **^c^** | 3.85±0.13 **^c^** | 34.95±1.11 **^d^** | 87.50±1.70 **^a^** | 29.90±0.60 **^a^** | 30.14±0.80 **^b^** | 15.95±0.63 |
| **G3A** | 11.94±0.30 **^b^** | 4.88±0.17 **^b^** | 38.14±1.08 **^c^** | 82.92±1.34 **^b^** | 28.10±0.59 **^a^** | 33.14±0.78 **^a^** | 14.3±0.80 |
| **G4A** | 13.58±0.38 **^a^** | 5.54±0.14 **^a^** | 41.06±1.20 **^b^** | 70.36±1.54 **^c^** | 22.46±0.48 **^c^** | 33.08±0.72 **^a^** | 14.48±0.78 |
| **G5A** | 11.08±0.31 **^bc^** | 4.44±0.16 **^bc^** | 42.8±1.22 **^b^** | 83.8±1.38 **^b^** | 24.22±0.70 **^b^** | 33.12±0.65 **^a^** | 14.12±0.82 |
| **G3B** | 11.625±0.35 **^b^** | 4.4±0.14 **^bc^** | 40.35±1.18 **^bc^** | 82.00±1.70 **^b^** | 28.75±0.64 **^a^** | 33.9±0.73 **^a^** | 15.72±0.82 |
| **G4B** | 13.18±0.42 **^a^** | 5.3±0.12 **^a^** | 40.08±1.05 **^bc^** | 69.26±1.63 **^c^** | 22.06±0.61 **^c^** | 33.44±0.69 **^a^** | 15.14±0.94 |
| **G5B** | 11.78±0.37 **^b^** | 4.68±0.15 **^b^** | 40.2±1.01 **^bc^** | 84.8±1.46 **^b^** | 24.22±0.66 **^b^** | 33.62±0.70 **^a^** | 15.75±0.86 |
| **G6** | 13.20±0.44 **^a^** | 5.42±0.12 **^a^** | 42.78±1.10 **^b^** | 80.43±1.59 **^b^** | 23.07±0.57 **^bc^** | 32.12±0.62 **^a^** | 15.68±0.77 |

Data presented as means ± SEM. The significant change was at p < 0.05. Means within the same column, which carry different superscript letters (a, b and c), are

significantly different (p < 0.01).

Supplementary Table 4. leukocytic cell count in different experimented groups

| Group | Total leukocytes  (10^3^/µl) | Neutrophil count  (10^3^ /µl) | Lymphocytic count  (10^3^ /µl) | Monocytes  (10^3^ /µl) | Basophil  (10^3^ /µl) | Eosinophil  (10^3^ /µl) | Platelets count  (10^3^ /µl) | MPV  (fl) |
| --- | --- | --- | --- | --- | --- | --- | --- | --- |
| **G1** | 7.58±0.33 **^d^** | 48.2±1.10 **^d^** | 40.2±0.90 **^a^** | 9±0.43 | 1.4±0.24 | 1.2±0.22 | 340.4±9.34 **^c^** | 6.3±0.30 **^b^** |
| **G2** | 13.95±0.42 **^a^** | 60.5±1.45 **^a^** | 36±0.82 **^b^** | 10±0.50 | 1±0.12 | 1±0.21 | 445.5±10.90 **^a^** | 8.1±0.37 **^a^** |
| **G3A** | 8.82±0.37 **^c^** | 53.6±1.03 **^bc^** | 33.4±0.84 **^c^** | 10.6±0.39 | 1.6±0.13 | 0.8±0.11 | 358.6±9.45 **^c^** | 6.18±0.29 **^b^** |
| **G4A** | 10.9±0.44 **^b^** | 53.00±1.12 **^c^** | 33.6±0.76 **^c^** | 10±0.56 | 1.6±0.11 | 0.8±0.10 | 380±9.91 **^b^** | 5.7±0.28 **^bc^** |
| **G5A** | 8.92±0.38 **^c^** | 54.8±1.14 **^bc^** | 35.6±0.78 **^b^** | 9±0.42 | 1.6±0.12 | 0.74±0.10 | 374.6±9.11 **^bc^** | 5.34±0.27 **^c^** |
| **G3B** | 9.85±0.40 **^bc^** | 52.25±1.09 **^c^** | 29.75±0.77 **^d^** | 9.25±0.44 | 1.5±0.14 | 0.75±0.10 | 383.5±9.60 **^b^** | 6.15±0.32 **^b^** |
| **G4B** | 9.08±0.38 **^c^** | 56.6±0.92 **^b^** | 35±0.80 **^b^** | 9±0.37 | 1.2±0.15 | 0.79±0.09 | 354.8±8.33 **^c^** | 6±0.25 **^b^** |
| **G5B** | 10.26±0.43 **^b^** | 52.00±0.98 **^c^** | 28.8±0.69 **^d^** | 9±0.31 | 1.2±0.10 | 0.69±0.11 | 382±8.94 **^b^** | 5.80±0.40 **^bc^** |
| **G6** | 8.52±0.36 **^c^** | 48.83±0.93 **^d^** | 39.67±0.74 **^a^** | 8.83±0.22 | 1.50±0.13 | 1.17±0.23 | 341.17±9.55 **^c^** | 6.12±0.37 **^b^** |

Data expressed as means ± SEM. The significant change was at p < 0.05. Means within the same column, which carry different superscript letters (a, b and c), are

significantly different (p < 0.01).
