# Supplementary figures and images for: Aspergillus awamori: potential antioxidant, anti-inflammatory, and anti-apoptotic activities in acetic acid-induced ulcerative colitis in rats
Source: Inflammopharmacology. 2024 May 20;32(4):2541–53. doi: 10.1007/s10787-024-01489-w (PMC11300502; doi:10.1007/s10787-024-01489-w)

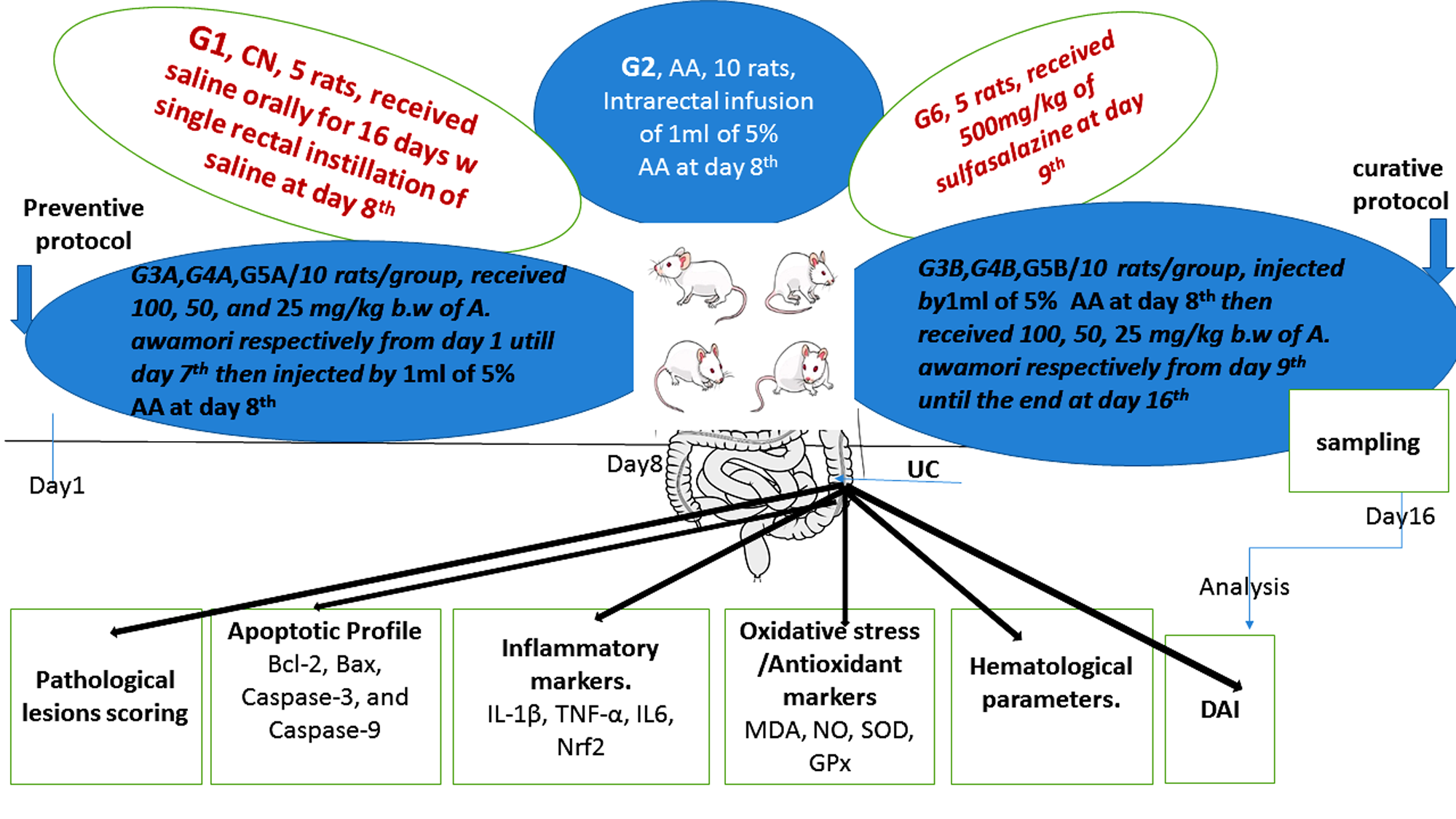

Supplement: Supplementary file 2 — Supplementary file2 (TIF 1032 KB) [file 10787_2024_1489_MOESM2_ESM.tif]
